# Sex comparisons of agonist and antagonist muscle electromyographic parameters during two different submaximal isometric fatiguing tasks

**DOI:** 10.14814/phy2.14022

**Published:** 2019-03-06

**Authors:** Sunggun Jeon, Xin Ye, William M. Miller

**Affiliations:** ^1^ Department of Health, Exercise Science, and Recreation Management The University of Mississippi University Mississippi

**Keywords:** Isometric, mean frequency, muscle fatigue, sex difference, surface electromyography, task dependency

## Abstract

To examine the task failure time of the force‐ and position‐based submaximal elbow flexion fatiguing tasks for both sexes, twelve men and eight women visited the laboratory for two separate experimental occasions. During the experiment, they pulled against a rigid restraint for the force task and maintained a constant elbow joint angle to support an equivalent inertial load for the position task. For both fatiguing tasks (50% of the isometric strength at the elbow joint angle of 135 degree), the task failure time, along with the surface electromyographic (EMG) amplitude and mean frequency (MNF) were measured. The average failure time was longer for the force task than that for the position task (sexes combined: 39.6 ± 16.6 sec vs. 33.9 ± 14.9 sec, *P* = 0.033). In addition, men were overall less fatigable than women (tasks combined: 42.0 ± 14.7 sec vs. 28.7 ± 10.3 sec, *P* = 0.020). The multiple regression analyses showed that the task failure time in women was solely predicted by the rate of change of the triceps EMG MNF. Thus, more fatigability of women in this study was likely due to the quicker fatiguing rate of the antagonist triceps brachii muscle. Different from most previous studies that have used 90‐degree elbow joint angle, the current 135‐degree joint angle setup might have created a situation where greater muscle activity from the related muscles (e.g., the antagonist) were required for women than for men to stabilize the joint, thereby resulting in a shorter task failure time.

## Introduction

Static and dynamic contractions are the two major modes of muscle action performed in daily physical activities. Determined by the muscle length change during a contraction, the dynamic mode can be categorized into shortening (concentric) and lengthening (eccentric) muscle actions. In contrast, without the changes in muscle length and joint angle, the static mode is often referred to as the isometric muscle action, primarily responsible for performing two tasks: attempting to shorten the muscle while joint motion is restricted, and maintaining a fixed posture/joint position while resisting the lengthening inertial imposed by an external load. With the similar mechanical (e.g., torque, force) requirements for both static tasks, previous studies have used different terms to describe them, such as isometric and isoinertial tasks (Buchanan and Lloyd 1995), and force and position tasks (Hunter et al. 2002). An interesting phenomenon regarding these two forms of static contraction is the task‐related difference in local muscle endurance, with longer task failure time performed during the force task, when compared to the equivalent inertial loaded position task (Hunter et al. 2002). Factors such as motor unit activities (Rudroff et al. 2010), overall muscle activation and perceived effort (Hunter et al. 2008), and the spinal reflex activities (Klass et al. 2008) have been examined and identified as the determinants for the task‐related difference. However, this task‐related difference tends to diminish and disappear during higher contraction intensities (Maluf et al. 2005; Rudroff et al. 2011). One of the potential mechanisms is thought to be related to the contraction intensity‐regulated muscle perfusion (Rudroff et al. 2011). Specifically, blood flow can be occluded at as low as 30% maximal voluntary isometric contraction (MVIC) (de Luca 1997), which results in the accumulation of metabolites. Thus, a higher contraction intensity is more likely to induce an enhanced accumulation of metabolites and greater pressor response, thereby playing a detrimental role on the task failure time during a fatiguing contraction.

In addition to the task factor, the sex factor has also been used to study the underlying mechanisms of muscle fatigue. Generally speaking, men are stronger than women, but less fatigue resistant during isometric fatiguing contractions at submaximal and maximal intensities (Hunter 2014). Besides the muscle fiber type difference (greater proportional area of type I fiber in women than in men) (Hunter 2014), other mechanisms also account for the sex‐related difference in fatigability. For example, when matched for strength, sex‐related difference in fatigability was absent during a fatiguing submaximal (e.g., 20% MVC) isometric contraction (Hunter et al. 2004). Additionally, muscle fatigue was similar for both sexes during the ischemic condition (Russ and Kent‐Braun 2003). These findings collectively revealed the potential mechanisms in sex‐related difference in fatigability, such as the differences in muscle fiber type, muscle mass, intramuscular pressure, and muscle perfusion between both sexes (Hunter 2014, 2016). Interestingly, limited research has directly compared potential sex‐related differences during both the force and position tasks. Previously, Rudroff et al. (2011) had both men and women in the experiment perform force and position tasks for four different contraction intensities, but the sex factor was not specifically mentioned or examined possibly due to the different purposes of that investigation.

The surface electromyography (EMG) technique has been used to monitor neuromuscular functions during muscle fatigue for years. Both temporal and spatial information of the EMG signals provides physiologic explanations of the changes in the neuromuscular system during an isometric fatiguing contraction (de Luca 1997). Specifically, an increase in the EMG amplitude during a submaximal sustained contraction could potentially indicate the increased neural drive to compensate for the contraction‐induced muscle fatigue, thus to meet the demand of the task. In addition to the prime mover agonist muscle, the gradually increased EMG amplitude have also been reported from the related antagonist and accessory muscles to explain the variation in task failure time (Hunter and Enoka 2003; Hunter et al. 2003; Rudroff et al. 2007, 2011). For example, under certain situations where the posture was manipulated (Rudroff et al. 2007), these muscles may play an important role limiting the task failure time. Besides the EMG amplitude, the EMG frequency parameter (e.g., mean frequency (MNF)) has been shown to be strongly associated with the muscle fiber conduction velocity (Lindstrom et al. 1970; de Luca 1984; Brody et al. 1991), and it is specifically sensitive to the changes in the accumulation of metabolites (e.g., H^+^ and K^+^) at the local intramuscular environment (de Luca 1997). Thus, the EMG MNF during a sustained submaximal isometric contraction also serves as a valuable fatigue indicator.

Previously, researchers (Merletti and Roy 1996; Troiano et al. 2008; Rudroff et al. 2011; Booghs et al. 2012; Beck et al. 2015; Carr et al. 2016) have used the rate of change in the EMG parameters to predict task failure time. For example, Booghs et al. (2012) reported the rate of changes in different muscles’ EMG amplitude as the best predictor for the elbow flexion task failure time during low and high contraction intensities. In addition, it has also been shown that the task failure time was highly correlated to both the decrement in high‐frequency spectral power and the increment in low‐frequency spectral power of the EMG signals (Beck et al. 2015); and the changes in EMG MNF can be used to predict time to failure during a medium‐intensity isometric elbow flexion fatiguing contraction (60% MVIC) (Carr et al. 2016). Interestingly, whether the rate of change in the antagonist muscle EMG MNF can contribute significantly to the task failure time during a sustained isometric contraction has rarely been examined. Considering the net torque around a joint is influenced by both agonist and antagonist muscle activities, the examinations of the antagonist muscle EMG parameters during a fatiguing contraction may provide additional important information (e.g., agonist antagonist imbalance) for evaluating sports performance and/or monitoring rehabilitation exercises.

Therefore, the primary purpose of this study was to examine the task failure times during both medium‐intensity submaximal (50% MVIC) force and position fatiguing tasks for both sexes. In addition, we measured the surface EMG parameters (amplitude and MNF) during the fatiguing contractions for both agonist and antagonist muscles, and calculated the rate of changes in these parameters, for the purpose of identifying if any specific parameter(s) would be the best predictor(s) for predicting the task failure time. We expected to see that the time to failure between both tasks may not necessarily differ, due to the relatively high intensity of the fatiguing contraction (Rudroff et al. 2011). However, the sex‐related factor may play a role influencing the muscle fatigability during different tasks. Considering the lower passive stiffness in shoulder and elbow joints in women than in men in general (Barnes et al. 2001; Eby et al. 2015), greater muscle activity from the related muscles (e.g., antagonist) may be needed for the stabilization purpose for the women, especially when they were performing the position task. Thus, the task failure time in women could specifically be shortened during the position task, when compared to the force task.

## Materials and Methods

### Experimental design

This study used a within‐subject crossover design to examine the task failure times and the EMG activities for the two fatiguing tasks (isometric elbow flexion force task vs. position task) in both sexes. In addition, the rate of change in EMG parameters (amplitude and MNF) versus task failure time relationships were also examined for the purpose of exploring and identifying potential predicting factor(s) for the local muscle endurance (task failure time). Three separate visits to the laboratory were required to complete this investigation, with a minimum of 48 h of rest provided between visits. The first visit was to familiarize the subjects with all experimental procedures. The second and third visits were experimental testing visits, which were conducted in a randomized order, where all dependent variables were examined in both force‐task and position‐task visits. All testing and interventions were performed with the dominant arm of the subjects based on their throwing preferences.

### Subjects

Twenty healthy and recreationally active men (*n* = 12, mean ± SD: age = 24 ± 4 years, height = 172.8 ± 5.7 cm, weight = 84.8 ± 12.1 kg) and women (*n* = 8, mean ± SD: age = 22.1 ± 3.1 years, height = 165.0 ± 3.9 cm, weight = 72.1 ± 11.8 kg) participated in this study. This investigation was approved by the University Institutional Review Board (protocol number: 18‐022). All experimental procedures were in conformity with the policy statement regarding the use of human subjects by the Helsinki Declaration of 1975, as revised in 2008. Before any experimental procedures, all subjects signed a written informed consent form and completed a health and exercise status questionnaire which indicated that they did not have neuromuscular, musculoskeletal, or cardiovascular diseases. Based on the questionnaire, all subjects were physically active. On average, they regularly performed aerobic exercise for 3 h, resistance exercise for 2 h, and recreational exercise for 2 h per week at least 6 months prior to this investigation. During the consenting process, all subjects were instructed to maintain their normal habits regarding dietary intake, hydration status, and sleep during the investigation. Extra effort was made to conduct testing at the same time of the day with the same arousal levels. In addition, the subjects were instructed to avoid any vigorous physical activity 72 h prior to the beginning of this investigation and during the entire experimental period.

### Procedures

**Figure 1:**
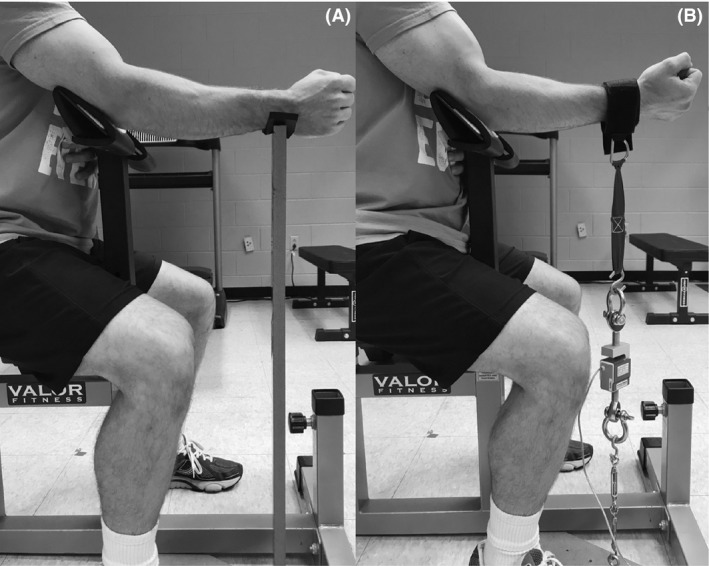
(A) A demonstration of elbow extension maximal isometric test; (B) A demonstration of elbow flexion isometric strength test.

During the familiarization visit (Visit 1), all subjects visited the laboratory to receive instructions and specifically to practice maximal isometric strength testing, as well as the two different submaximal fatiguing tasks. At least 48 h after the familiarization session, the subject returned to the laboratory for one of the experimental testing visits (force‐task visit or position‐task visit). Upon arrival, the subject was asked to sit in a preacher curl bench with the upright posture, with the dominant elbow placed onto the adjusted elbow support, and the nondominant arm placed on the abdominal region. The adjusted elbow support has a fixed angle of 135 degrees to the floor. After the setup, the maximal isometric elbow extension test was conducted. Specifically, the subject was asked to press the wrist down to an immovable wooden stand, which was firmly supporting the wrist of the dominant arm (Fig. 1A). Extra care was taken to ensure the wooden stand height was adjusted so the forearm was parallel to the floor, and with the exact elbow joint angle of 135 degrees. With the neutral hand position, 3 sets of 3‐sec maximal elbow extensions were performed. Instead of measuring the force value, the signal with the highest 1‐sec EMG root mean square (RMS) value was selected for normalizing later triceps brachii EMG parameters. After the elbow extension test, the investigator placed the subject's wrist into a cuff, which was connected to one end of a force transducer (Model SM‐500; Interface, Scottsdale, Arizona, USA), with the other end connected to an immovable board mounted to the floor (Fig. 1B). With the hand supinated, the subject performed 3 sets of 5‐sec MVICs, with the highest MVIC value selected as the elbow flexion isometric strength. Warm up sets were performed before both strength tests, and at least 1‐min of rest interval was provided between consecutive maximal contractions.

**Figure 2:**
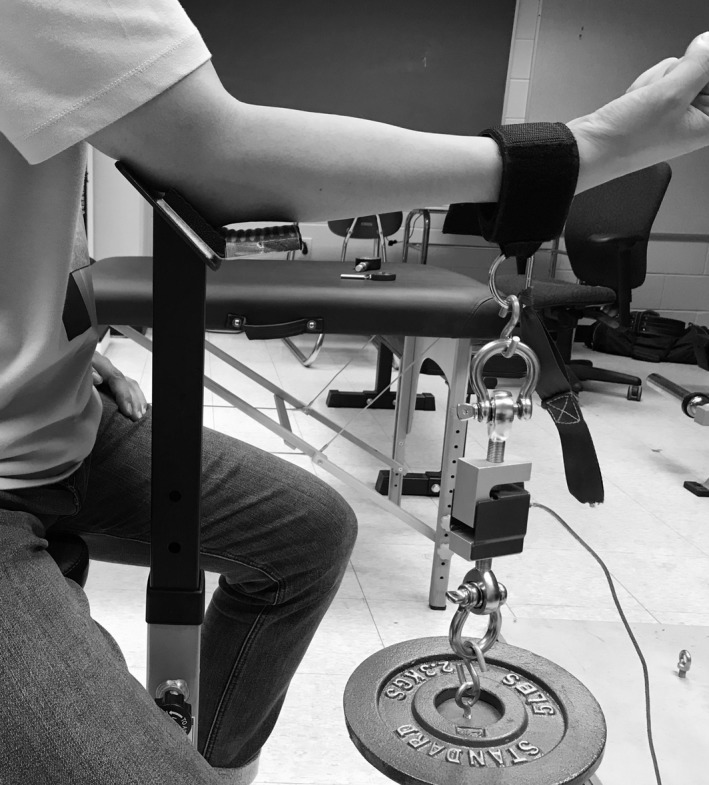
A demonstration of the elbow flexion position task.

At least 5 min following the MVICs, the measurement of task failure time was conducted for the designated fatiguing task. During the force‐task, the subject was asked to contract against the force transducer at the intensity of 50% MVIC until he/she could not reach this force level. During the position‐task, the subject was asked to maintain the forearm position as long as he/she could with a precalculated external load attached to the wrist cuff. The pre‐calculated external load was equivalent to the force of 50% MVIC. To closely monitor the forearm position, a small v‐shaped steel hinge with a fixed 135‐degree angle was attached to the elbow support right underneath the elbow joint (Fig. 2). Thus, the forearm would reach the horizontal part of the hinge even with a slight extension. During this task, verbal instructions from the investigator were provided to the subjects to maintain the 135‐degree elbow joint angle (with forearm at the horizontal position): if the forearm reached the hinge, the investigator would then immediately notify the subjects so the forearm could be pulled back to the horizontal position. Both target force and real‐time force were shown on the monitor, and the task failure criteria was the real time force below 50% MVIC for 3 sec or failing to maintain the forearm position for 3 sec. Strong verbal encouragement was provided by the investigators during the entire fatiguing contraction.

### Measurements

#### Force

The force produced during all the maximal and submaximal elbow flexion isometric contractions was detected by the tension applied to a force transducer (Model SM‐500; Interface, Scottsdale, AZ). The maximal isometric contractions were performed to set the required force during the sustained task (elbow flexion) and to normalize EMG (elbow flexion and extension). Specifically, the isometric strength force was quantified from the highest 1‐sec portion of the 5 sec MVIC. The force signal was sampled at 1000 Hz, and digitized with a 12‐bit analog to digital converter (Model USB‐6259; National Instruments, Austin, TX).

#### Surface EMG acquisition and data analyses

During all maximal and submaximal isometric contractions, surface EMG signals were recorded through the bipolar surface electrodes (DE 2.1 Single Differential Surface EMG sensor, 5‐mm interelectrode distance, Delsys, Inc., Natick, MA). Specifically, these sensors were attached over the biceps brachii and the long head of the triceps brachii muscle bellies based on the recommendations from SENIAM (Hermens et al. 1999). The reference electrode (Model USX2000; Axelgaard, Fallbrook, CA) was placed on the seventh cervical vertebrae (C7). Prior to any electrode placements, the investigator shaved and cleaned the skin surface with rubbing alcohol. And medical tapes were used to firmly fixate the electrodes on the skin sites. All analog bipolar EMG signals were collected and amplified (gain = 1000) with a modified Bagnoli 16‐channel EMG system (Delsys, Inc., Natick, MA) and filtered with high and low pass filters set at 20 and 450 Hz, respectively. The filtered signals were then digitized at a sampling rate of 20,000 Hz with a 12‐bit analog‐to‐digital converter (National Instruments, Austin, TX). For both fatiguing contractions, we selected the first (Begin), middle (Mid), and the last (End) 3‐sec windows of each EMG signal for further analyses. The amplitude of each selected window of each EMG signal was calculated as the RMS. For the EMG MNF analyses, the EMG signal was first filtered with a Hamming window, and the Discrete Fourier Transform (DFT) algorithm was used to derive the EMG signal into the power spectrum. The MNF of the spectrum was lastly calculated based on the equation described by Kwatny et al. (1970). All RMS and MNF values were normalized as percentages of the EMG values from the isometric strength testing trial that generated the highest force during each experimental visit.

### Statistical analyses

The maximal isometric strength values for the dominant elbow flexors among three visits (familiarization visit vs. force‐task visit vs. position‐task visit) were reliable, with *r* = 0.97 for the intraclass correlation coefficient model (3, 1) (ICC_3,1_) (Weir 2005) and no significant difference between experimental visits. In addition, the ICCs for the absolute EMG amplitudes and MNFs during Visits 2 and 3 were 0.72 and 0.85, respectively, with no significant differences between experimental visits.

A two‐way mixed factorial (sex [men vs. women] × condition [force vs. position]) analysis of variance (ANOVA) was used to examine the maximal elbow flexion isometric strength values between the two experimental visits for both sexes. To compare the task failure times for fatiguing contractions, a two‐way mixed factorial (sex [men vs. women] × condition [force vs. position]) ANOVA was used. In addition, separate three‐way mixed factorial (sex [men vs. women] × condition [force vs. position] × time [Begin vs. Mid vs. End]) ANOVAs were used to examine the normalized EMG amplitude and MNF throughout the entire fatiguing contraction between the two tasks for both sexes. When appropriate, the follow‐up test included one‐way repeated ANOVA, independent samples *t*‐tests, and paired samples *t*‐tests with Bonferroni adjustments (only applicable to the meaningful comparisons). For the rate of change in EMG parameters over time (from Begin to Mid to End), bivariate regression analyses were used to detemine the linear slope coefficients of the rate of change in absolute EMG amplitude (*μ*V/sec) and MNF (Hz/sec) for each subject during each fatiguing condition. Bivariate correlations were then conducted between the resulting linear slope coefficients from these regression analyses and the task failure time values. Lastly, separate stepwise multiple regression analyses were used to determine if the task failure time during different fatiguing tasks for each sex could be predicted by any single or the multiple linear slope coefficients of the rate of change in the biceps EMG amplitude, the biceps EMG MNF, the triceps EMG amplitude, and the triceps EMG MNF. The collinearity diagnostics from each regression analysis suggested that all variables in the regression model are independent (condition index < 15). All statistical tests were conducted using statistical software (IBM SPSS Statistics 22.0, IBM, Armonk, NY) with alpha set <0.05. In addition, effect sizes were calculated using Cohen's *d*, with 0.20, 0.50, and 0.80 as small, medium, and large effect sizes, respectively (Cohen 1992). All data are reported as mean ± standard deviation (SD) in the text and tables, and displayed as mean ± standard error of the mean (SE) in the figures.

## Results

### Isometric strength and actual force production during the fatiguing tasks

The two‐way ANOVA indicated no sex × condition interaction (*P* = 0.682) but there was significant main effect for sex (*P* < 0.001). After collapsing across condition, the isometric strength values were significantly greater for men than for women (mean ± SD: men vs. women = 382.0 ± 101.7 N vs. 189.0 ± 29.4 N, *P* < 0.001; *d* = 2.37). For the actual force produced during both fatiguing tasks, the men on average exerted 47.72% and 47.99% of the MVIC during force and position tasks, respectively. And the actual force produced by women during force and position tasks were 47.00% and 49.52% of the MVIC, respectively.

### Task failure time

Table 1 listed the task failure time values for both men and women during each fatiguing task. The two‐way ANOVA indicated no interaction (*P* = 0.149) but significant main effects for sex (*P* = 0.040) and condition (*P* = 0.036) for the task failure time. Specifically, men generally showed longer task failure time (with both tasks combined) than women did (men vs. women = 42.0 ± 14.7 sec vs. 28.7 ± 10.3 sec, *P* = 0.020; *d* = 1.01), and the force task generally displayed longer failure time (with both sexes combined) than position task did (force task vs. position task = 39.6 ± 16.6 sec vs. 33.9 ± 14.9 sec, *P* = 0.033; *d* = 0.35).

**Table 1:** Time to task failure values (seconds) and effect size (Cohen's *d*) values through comparisons for both men and women during both fatiguing tasks

	Men (*n *=* *12)	Women (*n* = 8)	Cohen's *d*
Force task	43.1 ± 18.3	34.1 ± 13.0	0.54
Position task	40.9 ± 13.7	23.4 ± 9.9	1.42
Cohen's *d*	0.13	0.93	

Values are given as mean ± SD.

### EMG parameters during fatiguing contractions

#### Agonist (Biceps Brachii)

The results from the 3‐way ANOVA indicated no significant three‐way interaction (*P* = 0.529), but there were significant sex × time interaction (*P* = 0.021) as well as main effects for sex (*P* = 0.022) and time (*P* < 0.001) for the normalized EMG amplitude of the biceps brachii. The follow‐up one‐way repeated measures ANOVA showed significant differences in EMG amplitude (Begin vs. Mid vs. End) for both men (*P* < 0.001) and women (*P* < 0.001), demonstrating gradually increased values across time. In addition, separate independent samples *t*‐tests showed that the normalized EMG amplitude was only significantly different at the End of the fatiguing contractions between sexes (men vs. women: 73.2 ± 23.7% vs. 101.4 ± 13.9%, *P* = 0.007, *d* = 1.38, Table 2).

**Table 2:** Normalized EMG amplitude (%) for the biceps brachii during fatiguing contractions between men and women

Variable	Men (*n *=* *12)	Women (*n *=* *8)	*P*	Cohen's *d*
Begin	58.2 ± 18.9	65.2 ± 10.4	0.054	0.43
Mid	72.0 ± 19.8	85.3 ± 15.6	0.129	0.73
End	73.2 ± 23.7	101.4 ± 13.9	0.007	1.38

Values are given as mean ± SD. *P < 0.05, sex × time two‐way interaction (*P* = 0.021), collapsed across conditions.

For the normalized EMG MNF, the 3‐way ANOVA showed no significant three‐ *(P* = 0.169) or two‐way interactions (time × gender: *P* = 0.786, condition × gender: *P* = 0.530, time × condition: *P* = 0.255), but there was a main effect for time (*P* < 0.001). After collapsing across sex and condition, the follow‐up pairwise comparisons showed that the EMG MNF was significantly greater at the Begin compared to at the Mid, greater at the Begin compared to at the End, and greater at the Mid compared to at the End of the fatiguing contractions.

#### Antagonist (Triceps Brachii)

The results from the 3‐way ANOVA indicated that there was no significant three‐way interaction (*P* = 0.935), but there were significant sex × time interaction (*P* = 0.007) as well as main effects for sex (*P* = 0.027) and time (*P* < 0.001) for the normalized EMG amplitude. The follow‐up one‐way repeated ANOVA showed that there were significant differences in EMG amplitude (Begin vs. Mid vs. End) for both men (*P* = 0.004) and for women (*P* < 0.001), demonstrating gradually increased values through time. In addition, separate independent samples *t*‐tests showed that the normalized antagonist EMG amplitude was significantly greater at the Begin, the Mid, and the End of the fatiguing contractions for women than those for men (Table 3).

**Table 3:** Normalized EMG amplitude (%) for the triceps brachii during fatiguing contractions between men and women

Variable	Men (*n* = 12)	Women (*n* = 8)	*P*	Cohen's *d*
Begin	11.4 ± 8.4	21.9 ± 12.8	0.041	1.01
Mid	15.6 ± 12.7	29.9 ± 15.6	0.037	1.03
End	17.3 ± 12.8	34.7 ± 16.5	0.016	1.21

Values are given as mean ± SD. *P < 0.05, sex × time two‐way interaction (*P* = 0.007), collapsed across conditions.

**Figure 3:**
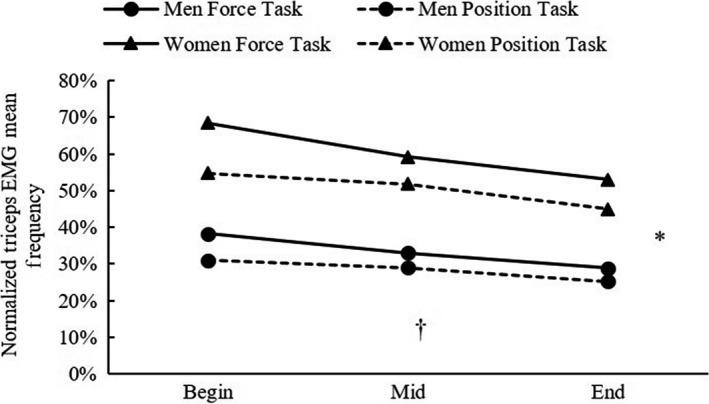
Normalized triceps brachii EMG mean frequency (MNF) during two different fatiguing contractions for both men and women. *Significant main effect for condition (force task vs. position task). ^†^Significant main effect for time (beginning (Begin) vs. middle (Mid) vs. last (End)).

For the normalized EMG MNF, the 3‐way ANOVA showed no significant three‐way interaction (*P* = 0.521). However, there were significant condition × time interaction (*P* = 0.002) as well as main effects for condition (*P* = 0.046) and time (*P* < 0.001). After collapsing across the sex, the follow‐up one‐way repeated measures ANOVAs showed significant differences in EMG MNF (Begin vs. Mid vs. End) for both force (*P* < 0.001) and position (*P* = 0.009) tasks, demonstrating gradually decreased values across time. In addition, separate paired samples *t*‐tests showed that the normalized EMG MNF was significantly greater at the Begin (*P* = 0.009) and the Mid (*P* = 0.044) of the force task than those of the position task (Figure 3).

### Contributing factors of the task failure time

Table 4 shows the results from the bivariate regression analyses of EMG parameters over time. Table 5 shows the results of the correlations between the linear slope coefficients for the rate of change in the EMG parameters and the task failure time for both sexes during both fatiguing conditions.

**Table 4:** Linear slope coefficients and *r*‐square values from the bivariate regression analyses of the biceps brachii and triceps brachii EMG amplitude and mean frequency (MNF) over time during the force task and position task fatiguing contractions

	Biceps (Amplitude)	Biceps (MNF)	Triceps (Amplitude)	Triceps (MNF)
Force task				
Slope coefficient	6.03 ± 5.12	−0.63 ± 0.28	0.46 ± 0.32	−0.59 ± 0.39
r^2^	0.82 ± 0.20	0.89 ± 0.20	0.88 ± 0.15	0.91 ± 0.13
Position task				
Slope coefficient	5.52 ± 6.77	−1.01 ± 0.70	0.75 ± 0.97	−0.62 ± 0.51
*r* ^2^	0.62 ± 0.36	0.94 ± 0.09	0.80 ± 0.26	0.94 ± 0.09

Values are given as mean ± SD.

**Table 5:** The Pearson correlation coefficient (Pearson's *r*) of the correlation between the linear slope coefficients for the rate of change in the biceps and triceps EMG parameters (amplitude and mean frequency (MNF)) and the task failure time for both sexes during both fatiguing conditions

			Biceps (Amplitude)	Biceps (MNF)	Triceps (Amplitude)	Triceps (MNF)
Men	Force Task	Pearson's *r*	−0.604	0.517	−0.319	0.544
		*P*	0.037	0.086	0.312	0.068
	Position Task	Pearson's *r*	−0.488	0.716	−0.699	0.495
		*P*	0.107	0.009	0.011	0.102
Women	Force Task	Pearson's *r*	−0.342	0.480	−0.495	0.804
		*P*	0.407	0.228	0.213	0.016
	Position Task	Pearson's *r*	0.002	0.580	−0.741	0.941
		*P*	0.995	0.131	0.036	<0.001

Values are given as mean ± SD.

Lastly, the results from the stepwise multiple regression analyses indicated that for men during the force task, only the linear slope coefficient for the rate of change in biceps EMG amplitude contributed significantly to the prediction of task failure time (regression model: task failure time (sec) = −2.226(slope biceps EMG amplitude) + 54.406; *r* ^2^ = 0.365). However, the linear slope coefficient for the rate of change in the biceps EMG MNF became a significant predictor (task failure time = 11.548(slope biceps EMG MNF) + 52.748; *r* ^2^ = 0.512) for the failure time during the position task. For women during both force (task failure time = 62.664(slope triceps EMG MNF) + 68.374; *r* ^2^ = 0.588) and position (task failure time = 21.023(slope triceps EMG MNF) + 39.401; *r* ^2^ = 0.886) tasks, the only significant predictor for task failure time was the linear slope coefficient for the rate of change in the triceps EMG MNF.

## Discussion

This investigation aimed to use the surface EMG parameters (amplitude and MNF) to examine the potential task‐ and sex‐related differences during submaximal isometric elbow flexion fatiguing contractions. In addition, the rate of change in the EMG parameters of both agonist and antagonist muscles were calculated, for the purpose of predicting the task failure time. The main findings of this study were as follows: (1) the task failure time was generally longer in the force task than in the position task (both sexes combined), (2) men generally sustained a longer time than women did during the fatiguing contraction (both tasks combined), and (3) different from men, the only significant predictor for the task failure time for women was the slope coefficient of the rate of change in the antagonist muscle EMG MNF during both submaximal fatiguing tasks.

The comparison of failure times between different fatiguing tasks (e.g., force vs. position tasks) have been extensively investigated (Hunter et al. 2002, 2008; Griffith et al. 2010), where a variety of factors (e.g., muscle group, contraction intensity, age, sex) were examined. Generally speaking, muscle fatigue is task dependent, with the task failure time tends to be shorter during the position task when compared with the force task (Hunter et al. 2002; Griffith et al. 2010; Baudry et al. 2011; Lauber et al. 2012). The current investigation showed a similar result in general (with men and women combined: force task vs. position task = 39.6 ± 16.6 s vs. 33.9 ± 14.9 s, *d* = 0.35). When examining submaximal isometric fatiguing exercise, an important factor that needs to be mentioned is the intensity used during the fatiguing tasks. Previous studies comparing the force task and position task at different intensities (Maluf et al. 2005; Rudroff et al. 2011; Booghs et al. 2012) showed that the task failure times were likely to differ when the sustained contractions were performed with a relatively low intensity (e.g., equal or less than 30% MVIC), but the difference tended to diminish with a higher intensity (e.g., greater than 45% MVIC). Rudroff et al. (2011) suggested that the contraction intensity‐related difference was likely due to the muscle perfusion factor during an isometric fatiguing contraction. Specifically, the contraction‐induced intramuscular pressure directly influences the rate of blood flowing out of the muscle. At relatively low intensities, occlusion of the blood flow progressively occurs, whereas at relatively high intensities, blood flow occlusion can occur at the onset of the contraction, which may lead to a rapid accumulation of metabolites in the active muscles. With the accumulation of the metabolites, the Group III and IV muscle afferents can be activated, inhibiting the nerve drive from the central nervous system (Amann 2012), thus to contribute to muscle fatigue (Rudroff et al. 2011; Booghs et al. 2012). Therefore, for this investigation with the subjects contracting at their 50% MVIC, the contraction intensity might still not be high enough to mask the failure time difference between the two fatiguing tasks. Interestingly, it is important to point out that this finding was likely affected by the large effect imposed by women (*d* = 0.93), rather than that by men (*d* = 0.13, Table 1). Both Rudroff et al. (2011) and Booghs et al. (2012) had female participants, but the potential influence of the sex factor on task failure time during the different fatiguing tasks were not examined or reported.

Perhaps the most interesting finding from this investigation is the result of the sex‐related difference in fatigability, which conflicts with the majority of the previous studies that have reported more fatigue resistant in women than in men (Hunter 2014). Men in this study sustained about 13 sec longer (both tasks combined) than women did. As expected, the results from the agonist and antagonist EMG parameters demonstrated consistent increases and decreases in the amplitude and frequency values during the fatiguing contractions, respectively. For the agonist EMG amplitude, both values of men and women gradually increased over time, but a sex‐related difference was found at the end portion of the fatiguing contractions, where agonist EMG amplitude in women increased more rapidly and reached to a greater value than that of men. In addition, our results also showed greater antagonist muscle activities at each stage of the fatiguing contraction in women than that in men. Collectively, these EMG amplitude responses might partially explain the sex‐related difference in task failure time, where a greater demand of the antagonist muscle activity was shown in women during the fatiguing contraction. More importantly, the correlations between the linear slope coefficient of the rate of change in the EMG parameters and the task failure time, along with the predicting factors from the multiple regressions explained the sex‐related difference in fatigability. Specifically, the predicting factors for failure time in men were the slope coefficient of the rate of changes in the agonist EMG parameters, which were in agreement with previous studies (Merletti and Roy 1996; Troiano et al. 2008; Beck et al. 2015; Carr et al. 2016). Interestingly, for both tasks performed by the women, the task failure times were exclusively predicted by the rate of change in the antagonist MNF. Thus, in this investigation, the antagonist EMG MNF, rather than the EMG amplitude, seemed to serve a better predictor of task failure time for women.

The antagonist muscle is responsible for maintaining posture, as well as stabilizing the related joint during a sustained contraction (Griffith et al. 2010). The net force produced by a certain joint is the difference between the amount of force generated by the agonist and by the antagonist. Thus, a quicker decline in the antagonist muscle EMG MNF is an indication of a faster fatiguing rate of triceps muscle, which could have the required greater antagonist muscle activation, thereby further requiring greater agonist muscle activity to meet the requirement of the net force. Therefore, the shorter failure time in women was likely due to an overall greater demand in agonist and antagonist muscle activities around the elbow joint. While it is difficult to pin down the exact factor(s) causing the quicker decline of triceps EMG MNF in women, we have proposed a possible explanation. Comparing to most of the previous studies where the elbow joint angle of the elbow flexion exercise was 90 degrees (Hunter et al. 2002, 2004; Mottram et al. 2005; Klass et al. 2008; Rudroff et al. 2010, 2011; Baudry et al. 2011; Booghs et al. 2012; Williams et al. 2014), our more extended (135‐degree) elbow joint provided a slightly different position. In addition to the obvious biomechanical changes, potential changes in the muscle activation patterns of the synergistic muscles (e.g., deltoid muscles, rotator cuffs) might also have occurred (Rudroff et al. 2007), requiring more shoulder and/or elbow joint stability at this position. Since women generally demonstrate lower passive stiffness in shoulder and elbow joints than men do (Barnes et al. 2001; Eby et al. 2015), a greater level of muscle activity from the related muscles around might be required for the purpose of stabilization when performing the fatiguing contractions at the current setup. This, therefore, could have resulted in shorter task failure time in women.

This investigation showed a few novel findings regarding the sex‐ and task‐related differences in time to task failure. However, it is necessary to be cautious when interpreting the findings from the study. Specifically, a major limitation includes the sample size, especially the relatively low number of female participants (*n* = 8). With this sample size, the results from the multiple regression analyses could have been less stable. In addition, our effect size calculations (Table 1) for the task failure time suggested that the women tended to fatigue quicker during the position task when compared to that during the force task (*d* = 0.93), and the position task seemed to play a major role (*d* = 1.42) differentiating the sex‐related difference in fatigability. However, the sex × condition interaction was not shown according to the statistics. It is possible that lack of the interaction for the task failure times might have been due to the low number of female subjects in this study. Besides the sample size, it is also worth pointing out that for our proposed explanation (women's less stable shoulder and elbow joints at the extended elbow joint angle) that could potentially be responsible for the shorter task failure time in women, this study did not collect related data or evidence to support such mechanism. Thus, to examine the antagonist and synergist muscle roles, fatigue studies can be conducted to examine task failure time with/without these muscles being supported/isolated. In addition, fatiguing tasks with different joint angles can also be performed to examine the roles of the related muscles (agonist, antagonist, and synergist) on fatigability.

In conclusion, both task and sex factors influenced the task failure time during elbow flexion fatiguing contractions at the intensity of 50% MVIC. Specifically, the failure time was generally (sex combined) longer in the force task than in the position task. In addition, men were generally (fatiguing task combined) more fatigue resistant than women during the fatiguing tasks. The task failure time in women was exclusively predicted by the rate of change in the antagonist muscle EMG MNF, which was likely to account for the sex‐related difference in fatigability. An explanation for the discrepancy regarding the sex‐related difference in fatigability between previous studies and ours is possibly due to the more extended elbow joint position in this investigation. With potentially less stable shoulder and elbow joints, women showed a faster fatiguing rate in the antagonist muscle. Thus, future research should be directed to examine the related synergistic and antagonist muscle activation patterns during stable vs. unstable positions or postures for both sexes.

## Conflict of Interest

The authors declare that they have no conflict of interest.
